# Towards an Automated Computational Workflow to Assess Primary Stability in Total Hip Arthroplasty

**DOI:** 10.3390/bioengineering12070723

**Published:** 2025-06-30

**Authors:** Massimiliano Mercuri, Enrico Toccaceli, Xiaoshu Sun, Giuseppe Marongiu, Marco Viceconti, Antonino Amedeo La Mattina, Cristina Curreli

**Affiliations:** 1 Department of Industrial Engineering, Alma Mater Studiorum—University of Bologna, 40136 Bologna, Italy; massimiliano.mercur2@unibo.it (M.M.); enrico.toccaceli2@unibo.it (E.T.); xiaoshu.sun@unibo.it (X.S.); marco.viceconti@unibo.it (M.V.); 2 Department of Surgical Sciences, University of Cagliari, 09042 Monserrato, Cagliari, Italy; giuseppe.marongiu@unica.it; 3 Policlinico Universitario Duilio Casula, Azienda Ospedaliero Universitaria di Cagliari, 09124 Cagliari, Italy; 4 Medical Technology Lab, IRCCS Istituto Ortopedico Rizzoli, 40136 Bologna, Italy; cristina.curreli@ior.it

**Keywords:** finite element modeling, total hip arthroplasty, primary stability, high-performance computing, preoperative planning

## Abstract

Total hip arthroplasty is one of the most common and rapidly growing surgical procedures, with over one million cases performed annually in the United States. Despite high success rates, revision surgeries remain a significant concern due to complications such as aseptic loosening, often resulting from inadequate primary implant stability. This study presents an automated computational framework that integrates three-dimensional preoperative planning and finite element modeling to predict the primary stability of hip implants. Data obtained from the virtual surgery phase are used to generate subject-specific finite element models, which are executed on high-performance computing systems. The simulation evaluates implant stability by analyzing the contact interaction between the bone and the implant. The pipeline is demonstrated using data from the open-source HFValid collection and a commercial implant. Automation substantially reduced the time required to set up simulations, improving the efficiency on high-performance infrastructure. This integrated computational approach bridges the gap between biomechanical modeling and clinical decision-making and can serve as a preclinical tool for identifying personalized implant strategies and for conducting large-scale virtual cohort studies.

## 1. Introduction

Primary total hip arthroplasty (THA) is among the top five of modern medicine’s most common and faster-growing surgical procedures. In 2023, more than 1 million THAs were performed in the U.S., according to the American Joint Replacement Registry [1], representing a 10% increase from the previous year [2]. This number is expected to increase exponentially by the end of 2030 [3,4]. Despite its excellent clinical performance, with a 10-year survival rate above 90% in developed countries [5,6], the revision rate remains a significant burden, with an approximately 4–6% risk of failure at six- to ten-year follow-ups, carrying significant health and economic costs; from 2006 to 2015, the reported average cost per revision procedure was USD 75,037 [7]. Aseptic loosening is the primary cause of THA revision, followed by instability (i.e., dislocations), infections, and wear [6,8]. Aseptic loosening in modern cementless implants is primarily caused by a lack of primary stability, which can increase initial bone–implant micromotion, leading to fibrous connective tissue formation at the implant interface and eventually implant migration into the femoral canal during the initial postoperative period [9,10]. Micromotion larger than 150 μm prevents osseointegration [10,11,12] and axial migration over 1.5 mm in the first two years is linked to revision rates as high as 50% [13].

Finite element (FE) modeling is an established method to investigate the impact of various factors on primary implant stability in both individual subjects and diverse cohorts. These factors include the impact of the collar [14], neck lateralization [15], inter-subject variability [15,16], surgical variability [17,18], and variations in hip joint loading due to different motor tasks and subject-specific motor strategies [19]. In these previous studies, except for the latest, the final implant position and design have been selected with an in-house developed script that automatically finds the best options based on femoral anatomical parameters such as femoral shaft axis, neck axis, femoral-shaft angle or caput-collum–diaphyseal (CCD) angle, neck length, and femoral offset. Variability in surgical outcomes has been investigated by altering this defined optimal position. Automatic tools can effectively investigate the anatomical compatibility of a new implant in the early phases of the design process. Still, they do not consider surgeon experience and knowledge [20] when used in surgical pre-operative planning.

In the last decade, different solutions have been developed for THA planning based on patient-specific Computed Tomography (CT) images, clearly demonstrating the advantages of three-dimensional computer-based preoperative planning over traditional templates, such as conventional radiograph templates [21]. Compared to conventional techniques, 3D pre-operative planning tools accurately define anatomical features and predict component sizing, thus reducing the surgical time (less intra-operative guesswork) and implant inventory, and leading to a more cost-effective THA [22]. When considering stem positioning, many planning tools have incorporated range-of-motion simulations [23,24,25], surgical access and muscle retraction [25], or spectrum maps to assess the contact area of the stem and femoral intra-medullary cavity [26,27,28]. Still, no one has incorporated a biomechanical model to investigate the primary stability. Reggiani et al. [29] were the first to develop a subject-specific finite element model using pre-operative planning software for stem size and position selection, predicting the primary stability with sufficient accuracy when compared to an equivalent in vitro experiment. However, the procedures for automatically extracting and setting up such models were not integrated into the planning software.

This study aims to present a comprehensive computational framework that includes all the essential steps required to create a subject-specific FE model to predict the primary stability of THR. It combines the advantages of 3D-based preoperative planning systems with biomechanically derived numerical analysis performed on high-performance computing platforms. Once the simulation model is validated, this framework could serve as a preclinical tool for testing new stem designs and assist in educating surgeons on implant selection and positioning. Additionally, it could be used to automatically create in silico trials to generate data supporting the performance of new devices considering a large virtual cohort of simulated patients. To describe the methodology and demonstrate the capabilities of the computational pipeline, an FE simulation was performed to assess the primary stability of a hip stem using data from the HFValid collection [30] and a commercial implant design from Adler Ortho.

## 2. Materials and Methods

### 2.1. Description of the Workflow

**Figure 1:**
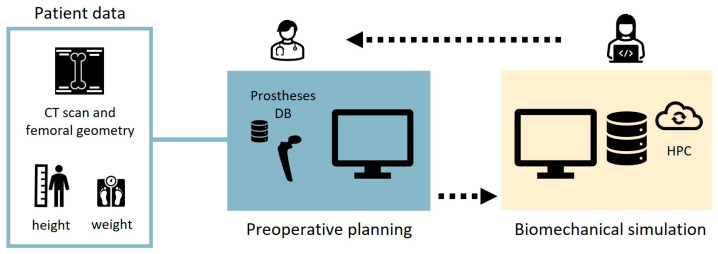
Workflow of the computational process. The surgeon selects the appropriate prosthesis for the patient via a multimodal display user interface and sends the output files to a remote operator. The operator prepares the simulation files for HPC resources and processes the results of the simulation.

A schematic representation of the computational workflow is presented in Figure 1. The process begins when the patient arrives at the hospital and undergoes a calibrated bilateral femoral CT scan. The scan covers a field of view extending from the proximal iliac crest to the distal femur condyles. Following the scan acquisition, clinicians load the DICOM images into the developed preoperative planning interface, named HiPlan, to perform virtual total hip arthroplasty planning on one or both sides using a hip implant database (*preoperative planning*). Once this virtual surgery is completed, output data saved in standard files are compressed in .zip format are transferred to a remote service laboratory, where a technician checks the data and submits the biomechanical model to a HPC cluster (*biomechanical simulation*). After the analysis is completed, the results are automatically processed, checked by the technician, and made accessible to clinicians for evaluation.

### 2.2. Preoperative Planning

**Figure 2:**
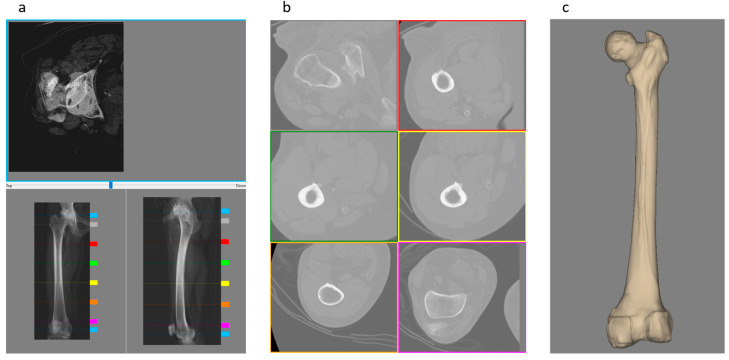
The multimodal display principles. (**a**) The XR-window where the CT dataset is represented as two orthogonal radiography projections: antero-posterior view (bottom left) and medio-lateral view (bottom right), together with a merged view (top) of two CT slices to visualize the femoral anteversion. The colored sliders at the edge of the XR-projection image are used to choose the CT slices for the anteversion representation (blue sliders) and (**b**) the six CT slices of the CT-window (sliders from gray to magenta); (**c**) the 3D-window, where the femoral bone is represented with a surface rendering.

The computational framework developed in this work begins with the preoperative planning phase for total hip replacement surgery through a user interface named HiPlan. This task was achieved through the implementation of a multi-modal display interface inspired by Hip-Op software [27,31]. The multimodal display interface is a visualization paradigm where anatomical objects are represented through multiple views, each one simulating a different imaging modality familiar to the medical professionals, the intended end users of the application. These imaging modalities, as shown in Figure 2, are represented in three windows named XR-window, CT-window, and 3D-window. The XR-window (Figure 2a) displays the anatomy as two orthogonal radiography projections, representing the medio-lateral and the antero-posterior views, which are artificially generated from the CT scan data. Additionally, the XR-window includes a combined view of two CT slices, a proximal and a distal slice, to provide information about the femoral anteversion. Six CT slices are shown simultaneously in the CT-window (Figure 2b); the user can choose the slices to visualize by using six colored sliders in the radiography views. The 3D-window (Figure 2c) shows the femur region with a surface rendering.

The HiPlan interface imports the CT volume dataset (stored in a DICOM folder, or as NRRD or NIfTI file) and, if available, the segmented femur shape (STL format), and the respective windows are generated showing radiography projections and the six CT slices, together with the 3D rendering. If femur segmentation is not provided, HiPlan performs threshold segmentation (multi-Otsu filtering technique) to extract an approximative femoral geometry for visualization purposes.

From one of the two XR views the user can perform on the femur relevant measurements that can affect the proximal stability and influence the prosthesis choice, such as neck–shaft angle, canal flare index, and/or Dorr score [32,33,34], using a specialized interactive tool.

Based on these measurements, the user can then select the most appropriate prosthetic model from a predefined (yet extensible) implant database, displayed across the multiple views for an easy comparison of possible alternative prostheses.

The pose (positioning and orientation) of each prosthetic model in the global space is controlled by a transformation matrix. By default, rotations are performed around the center of the implant bounding box, but the user can relocate the visual rotation center to any desired position. The implant can be translated and rotated either by mouse click-and-drag in the three views of the XR-window or by more precise interactive controls (text boxes). The user cannot move the prosthesis in the CT-window or the 3D-window, but can visualize the cross sections aligned with the CT slices in the CT-window, or adjust the rendering viewpoint (pan, zoom, and rotate) in the 3D-window.

Additionally, the osteotomy procedure is planned by rotating and translating a cubic shape in the XR-window to resect the femoral head and neck. The surgeon chooses the positions and orientation of the cube to accommodate anatomical variations.

The pre-operative planning of the total hip replacement surgery interface was developed in Python (version 3.10) using Qt/PySide6 (version 6.5.1.1) [35], Visualization Tool Kit (VTK, version 9.2.6) [36], and Insight Segmentation and Registration Toolkit (ITK, version 5.3) [37]. A TOML configuration file was employed to manage the structured data of the prostheses (e.g., types, sizes, and STL file paths). The output data from the preoperative planning step include (i) details about the prosthetic implant selected by the surgeon (i.e., model name, size, and type) and the final pose transformation matrices of both the prosthesis and the resection cube, saved in a standardized text file (TOML); (ii) the draft geometry of the femur, saved in a binary STL file; (iii) anonymized CT scan images converted to NRRD format; and (iv) patient-specific information, such as height and weight, saved in a standardized text file (TOML). All the output data are automatically compressed into a .zip file and copied to a job-specific folder with restricted access, where the technician can retrieve it to build the numerical model.

### 2.3. Biomechanical Simulation

**Figure 3:**
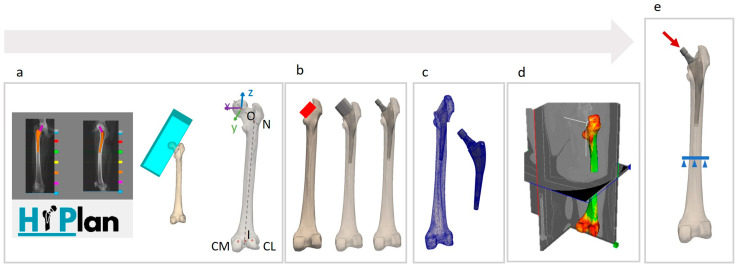
Complete automatic workflow for model preparation. (**a**) The stem size, type, and position, along with the resection cube, are obtained from the HiPlan pre-operative planning interface. Anatomical landmarks are also identified (O, center of femur head; N, intersection between femur canal midline and neck axis; I, intercondylar notch; Cl, lateral condyle; CM, medial condyle). (**b**) Boolean subtraction is performed to replicate the femoral head and neck resection, followed by the reaming procedures with the rasp. (**c**) The geometry of both the bone and the stem is meshed for simulation purposes. (**d**) Material properties are assigned element-wise, with local Young’s modulus depending on CT scan data. (**e**) Loading and boundary conditions are set: the distal femur is fixed and the hip reaction force is applied to the stem.

The biomechanical analysis includes different steps (Figure 3). The first (i.e., femoral geometry refinement) is performed on the operator workstation, while the others (i.e., Boolean subtractions, meshing, material property mapping, and FE simulation) are offloaded to a HPC cluster (Leonardo, CINECA, Bologna, Italy) with SLURM submission scripts to facilitate efficient resource management. Special attention was given in configuring virtual environments and managing software dependencies, utilizing dependency control tools and precompiled modules.

#### 2.3.1. Refinement of Femoral Geometry and Local Reference System

The process of building the biomechanical model begins with refining the segmentation of the femoral geometry from the CT images. This process can be performed manually or through automatic and semi-automatic unsupervised methods available in the literature. In the current workflow, a semi-automatic unsupervised method based on the graph-cut algorithm with manual correction has been adopted [38]. In addition, five anatomical landmarks needed to define the femur local reference system and loading conditions are manually identified on the bone surface (Figure 3a). These include the center of the femoral head (O), the intersection point between the curved femoral midline and the neck axis (N), the intersection point between the curved femoral midline and the cortical bone of the intercondylar notch (I), and the centers of the two half-circles representing the dorsal aspect of the lateral and medial femoral condyles (CL and CM) in the lateral view [39].

#### 2.3.2. Boolean Subtraction

An automatic Boolean subtraction technique, implemented using Python PyMeshLab library (version 2023.12.post2), is performed to reproduce the reaming activity during the intervention. The cubic shape positioned by the clinician using the pre-operative planning interface is used to mimic the femoral head and neck resection. The rasp corresponding to the selected stem is then positioned using the stem roto-translational matrix obtained from the pre-operative planning interface, and a Boolean subtraction between the resected femur and the rasp is performed.

#### 2.3.3. Meshing

The CAD model of the selected stem is placed into the generated femoral cavity by using its transformation matrix. The stem and the femoral geometries are meshed separately using Ansys ICEM CFD (release 2023 R2, Ansys Inc., Canonsburg, PA, USA) with the Octree method, adopting 3D linear tetrahedral elements and a default maximum edge length of 2 mm. Mid-side nodes are added to each edge of the tetrahedral elements using a script implemented in Ansys Mechanical APDL (release 2023 R2, Ansys Inc., Canonsburg, PA, USA) and the final meshes were converted into a 10-node quadratic tetrahedral meshes.

#### 2.3.4. Material Property Mapping

An in-house developed Python clone of Bonemat [40] is used to map CT densitometric data into a heterogeneous material mesh for the bone tissue, ensuring an accurate representation of patient-specific material properties [41]; in particular, Young’s modulus in the femur is assigned element-wise depending on the local Hounsfield Unit, while Poisson’s ratio was set to 0.3. The Apta-fix Adler stem, made of titanium alloy, was modeled as a homogeneous material with Poisson’s ratio of 0.3 and Young’s modulus of 115 GPa.

#### 2.3.5. FE Simulation

A preliminary subject-specific FE simulation was performed by using Ansys Mechanical APDL to demonstrate the feasibility of the workflow. The applied loading conditions replicate the maximum force on the hip joint during stair climbing [39], scaled according to the subject body mass. The force components were considered in the femur coordinate system, defined as follows. The origin is located at the center of the femoral head. The z-axis is directed along the line connecting N and I. The x-axis is perpendicular to the z-axis and parallel to the axis connecting C_L_ and C_M_ in the transverse plane. The z-axis points upwards, the x-axis points medially, and the y-axis follows the right-hand rule (Figure 3a). The hip contact force is applied to a pilot node defined at the center of the circular top surface of the stem neck. The pilot node is rigidly constrained to the proximal surface of the stem neck with a multipoint constraint approach. A fixed support is defined at the distal femur, 50 mm below the apex of the stem [42]. Surface-to-surface large sliding contact, with no interference fit and a coefficient of friction of 0.3, is applied along the entire length of the stem–bone interface to model the interaction between the stem and host bone. The Augmented Lagrangian algorithm was selected for the contact analyses considering the Gauss point detection method and a normal contact stiffness update at each iteration based on the allowable penetration and current mean stress of the underlying elements. Quantities of interest extracted as the main simulation outputs include contact sliding distance, contact pressure, penetration, and gap.

A CSV file containing the node connectivity of the contact region was also created to obtain a colored map distribution of the quantities of interest in an interactive web-based visualization, exported in HTML through the Python Plotly library (version 6.0.0).

### 2.4. Case Study

The complete workflow was tested on one patient from the HFValid Collection (female, age 72, height 154 mm, and weight 90 Kg), downloaded from the data repository link (https://amsacta.unibo.it/id/eprint/7277/, accessed on 3 February 2025). The database includes 101 calibrated CT scans, the corresponding femur segmentations, anatomical landmarks, and the patient’s age, height, and weight. The test implant database included stem geometric models of the prosthetic implants Apta-Fix (Adler, Milan, Italy) in STL format, sorted by implant name and size. Models of rasps were generated from the CAD of the stems by adding a cylindrical extension at the apex and an extrusion on the lateral–proximal part, using Ansys Spaceclaim (release 2023 R2, Ansys Inc., Canonsburg, PA, USA). The size of the stem and its pose inside the femur were defined by an expert orthopedic surgeon.

## 3. Results

### 3.1. Preoperative Planning

**Figure 4:**
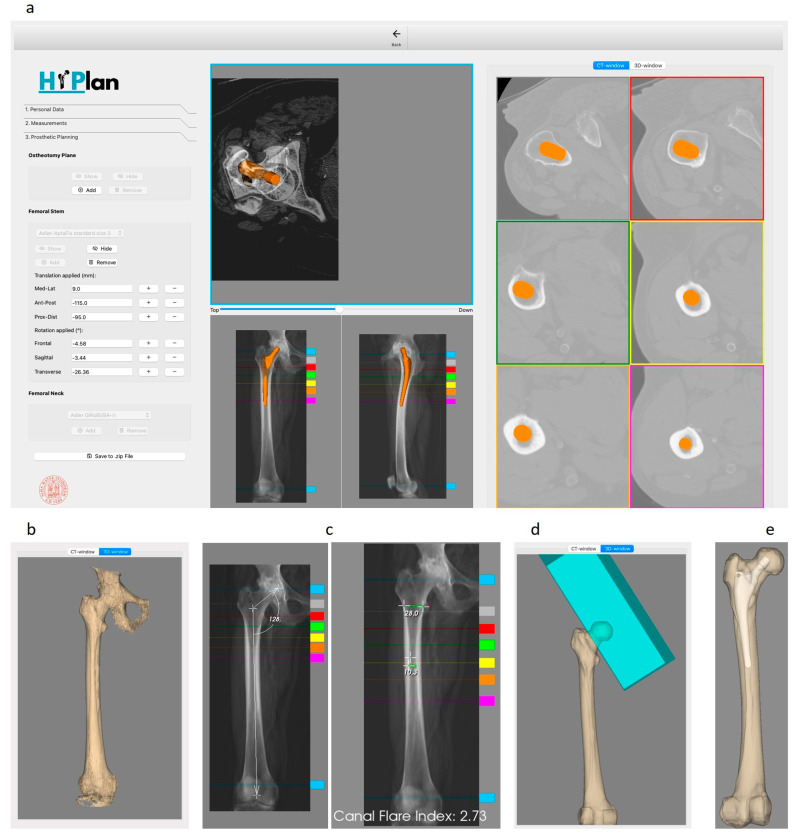
Main outputs of the preoperative planning step. (**a**) Developed preoperative planning interface and CT-window where the implant contour map is displayed across six slices. (**b**) Approximate segmentation computed when no external segmentation is imported. (**c**) Examples of femur neck–shaft angle and canal flare index measurements. (**d**) Results of the simulated osteotomy procedure and (**e**) final implant pose.

Figure 4 shows the developed preoperative planning interface and the implant positioning results obtained considering the selected case study. The Tab Manager guides the user through different steps (Figure 4a): the “Personal Data” tab displays patient information such as ID, height, weight, and whether one or both legs are considered for THA; the “Measurements” tab enables the user to measure key parameters for patient classification; the “Prosthetic Planning” tab allows for the positioning of the osteotomy block and implant. Using the “Add” button, a new osteotomy box is placed, which the user can translate or rotate using the multiple views in the XR-window. The box can be removed with the “Remove” button and can be temporarily hidden or made visible again with the “Hide” and “Show” buttons, respectively. The user can then choose the appropriate femoral stem from a combo box with the available options. In the XR-window, the stem can be translated or rotated around the center of the implant bounding box. Additionally, the center of rotation can be changed using the right mouse button in combination with the “C” key. For fine adjustments, the “Femoral Stem” box includes plus and minus buttons that allow translations of 2 mm and rotations of 2 degrees. The “Femoral Neck” box, which activates only when a modular femoral stem is chosen, allows the user to select a neck from the appropriate configuration catalogue. The software efficiently manages the complex relationship between the modular components, ensuring accurate positioning. In the CT-window, the implant contour map is displayed across six slices, selected via colored lines superimposed on the medio-lateral and antero-posterior radiographic images.

The approximate segmentation computed when no external segmentation is imported includes pelvic regions and the proximal tibial plateau (Figure 4b).

An example of femur measurements (e.g., the neck–shaft angle and the canal flare index) extracted interactively is shown is Figure 4c. Both measurements are taken from the antero-posterior synthetically generated X-ray image located at the bottom left of the XR-window. The neck–shaft angle is measured using a 2D VTK angle widget by placing three points on the image. The canal flare index is calculated as the ratio between two VTK distance widgets defined on the image: the first is placed by the user by selecting a segment 2 cm above the lesser trochanter, and the second defines the endosteal width.

Figure 4d,e displays the results of the simulated osteotomy procedure and the final implant pose defined by an expert orthopedic surgeon. Size 1 of the monolithic Apta-fix Adler stem was selected for the patient.

### 3.2. Biomechanical Simulation

**Figure 5:**
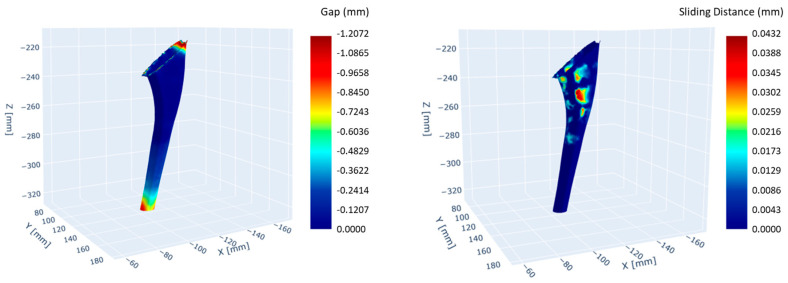
Interactive maps of gap and sliding distance in the contact area between implant and bone.

The automated Boolean subtraction process has enabled a faster simulation of the reaming activity during the intervention, reducing the time by 90% compared to the manual procedure. The biomechanical simulation was run on an HPC cluster, which further reduced the computation time by 65%. Some of the results obtained, including the contact gap and the sliding distance, are presented in Figure 5. The peak contact pressure was found to be around 28 MPa, while the sliding distance was less than 50 µm.

## 4. Discussion

In this work, an automated computational workflow for predicting the primary stability in total hip arthroplasty is introduced. The process begins with the acquisition of patient imaging, followed by the virtual implantation of the desired stem, which is performed by the surgeon using HiPlan, a preoperative planning software specifically developed for this context.

The preoperative software is based on a previous version of a Hip-Op software solution that integrates the multi-modal display principle [27,31]. Compared to Hip-Op, this updated solution introduces the capability to measure clinically relevant parameters related to the femoral morphology, such as the neck–shaft angle and canal flare index, both of which can influence the implant selection and survival rate [43]. Additionally, this software allows for the selection of either a modular or monolithic stem, enhancing surgical planning, particularly in revision total hip arthroplasty [44]. The user interface is specifically designed for integration into a biomechanical simulation, aiming to represent surgical procedures as accurately as possible. Consequently, the surgeon not only selects the type of implant, but also determines the resection plane for the femoral neck. The final output of the planning process is a zip file containing all the files and information needed to run the simulations in a standardized fashion.

The data are sent to a remote laboratory, where a trained engineer reviews the implant position, defines a local reference system to apply loading conditions, and submits the case for computation on HPC resources. Once the local reference system is defined, the Boolean subtraction simulating the reaming procedure during surgery is automatically launched, along with meshing and material mapping. This automation significantly reduces the need for manual operations and minimizes the time required to perform these tasks. Additionally, adapting the workflow to run on HPC resources using SLURM submission scripts improves resource management and further reduces the computation time.

Compared to similar workflows in the literature [14,16,17,18,29], this approach allows for full clinical integration with a clearly defined decision-making role for both the surgeon and the engineer. The implant choice, positioning, and resection plane are determined by the surgeon, rather than relying on a computational script that selects the best fit from an implant database based on the patient morphology. Additionally, the workflow is fully optimized for execution on HPC resources, with most manual operations reduced to simple job supervision, thus marking a significant innovation. The workflow can easily be extended to perform a Monte Carlo analysis with the aim of evaluating the consequences of the prosthesis insertion by varying the variables of influence (e.g., the prosthesis pose and size and the degree of osteoporosis of the patient).

However, the workflow was tested on a single patient and virtual implantation was performed by a single surgeon. Also, the model results have not been validated against experimental measurements. Further study should be carried out to evaluate subject variability on a larger cohort and inter–intra operator variability, as well as the model sensitivity to the input data and parameters. The simulation results must be thoroughly analyzed to establish credibility prior to any clinical use.

This work helps to bridge the gap between computational modeling and clinical decision-making, improving collaboration between engineers and surgeons while simplifying the planning process for total hip arthroplasty. By reducing the computational time and the manual interventions, the approach also moves toward real-time or near-real-time surgical simulations, which could support better preoperative planning and more personalized treatment strategies.

## Data Availability

Original CT scan data were obtained from the publicly accessible dataset HFValid (https://doi.org/10.6092/unibo/amsacta/7277) under the terms of the CC BY 4.0 International license. Additional data supporting the conclusions of this article will be made available by the authors on request.
